# The SmartHand transradial prosthesis

**DOI:** 10.1186/1743-0003-8-29

**Published:** 2011-05-22

**Authors:** Christian Cipriani, Marco Controzzi, Maria Chiara Carrozza

**Affiliations:** 1 The BioRobotics Institute, Scuola Superiore Sant'Anna, V. Piaggio, 34 - 56025 - Pontedera (PI), Italy

## Abstract

**Background:**

Prosthetic components and control interfaces for upper limb amputees have barely changed in the past 40 years. Many transradial prostheses have been developed in the past, nonetheless most of them would be inappropriate if/when a large bandwidth human-machine interface for control and perception would be available, due to either their limited (or inexistent) sensorization or limited dexterity. *SmartHand *tackles this issue as is meant to be clinically experimented in amputees employing different neuro-interfaces, in order to investigate their effectiveness. This paper presents the design and on bench evaluation of the SmartHand.

**Methods:**

SmartHand design was bio-inspired in terms of its physical appearance, kinematics, sensorization, and its multilevel control system. Underactuated fingers and differential mechanisms were designed and exploited in order to fit all mechatronic components in the size and weight of a natural human hand. Its sensory system was designed with the aim of delivering significant afferent information to the user through adequate interfaces.

**Results:**

SmartHand is a five fingered self-contained robotic hand, with 16 degrees of freedom, actuated by 4 motors. It integrates a bio-inspired sensory system composed of 40 proprioceptive and exteroceptive sensors and a customized embedded controller both employed for implementing automatic grasp control and for potentially delivering sensory feedback to the amputee. It is able to perform everyday grasps, count and independently point the index. The weight (530 g) and speed (closing time: 1.5 seconds) are comparable to actual commercial prostheses. It is able to lift a 10 kg suitcase; slippage tests showed that within particular friction and geometric conditions the hand is able to stably grasp up to 3.6 kg cylindrical objects.

**Conclusions:**

Due to its unique embedded features and human-size, the SmartHand holds the promise to be experimentally fitted on transradial amputees and employed as a bi-directional instrument for investigating -during realistic experiments- different interfaces, control and feedback strategies in neuro-engineering studies.

## Background

The hand is a powerful tool and its loss causes a severe psychological and physical drawback. Despite the significant impact of losing a hand, numbers of amputees requiring a prosthesis are too small to push manufacturers to innovate their products, so that both control interfaces and mechanisms have barely changed in the past 40 years [1]. The most technologically advanced prostheses are myoelectric ones: one or two degrees of freedom (DoFs) motorized hands (or hooks) are activated by antagonist residual muscle contractions where the electromyographic (EMG) signal is picked-up by surface electrodes in the prosthetic socket and processed to functionally open and close the hand (or pronate/supinate the wrist). These prosthetic hands, commercially available since the early 1970's and produced by different manufacturers (Otto Bock, Austria; RSL Steeper, UK; Motion Control, Utah; LTI, Massachusetts), are robust, weigh up to 600 g and are able to impart up to 100 N to objects due to their pincer-like shape. Low functionality, cosmesis and controllability have been considered as the main drawbacks for such devices [2] and surveys on their usage reveal that 30 to 50% of upper limb amputees do not use them regularly [3]. The lack of musculoskeletal and proprioceptive sensory feedback in myoelectric prostheses is one of the main reasons for their rejection [3]: a stump with intact sensory feedback fitted with a body-powered prosthesis (that transmits vibration and grasping force to the stump through the harness) is often more functional than a myoprosthesis with no purposely delivered sensory feedback [4]. Some of these drawbacks could be overcome by a product that recently entered the market. In July 2007 a Scotland based company, Touch Bionics, has launched a novel multi-articulated prosthesis: the i-LIMB hand. This is the first-to-market prosthesis with five individually-powered digits and a thumb abduction/adduction passive movement. Consequently the hand is capable of different grasping patterns, nevertheless it still uses a traditional two-input EMG system to simultaneously open and close all fingers. Over and above, no sensory feedback is delivered to the wearer.

The development of a more functional and naturally controlled prosthetic hand has been an active research field for decades and is still one of the big research challenges in rehabilitation, for which a tight collaboration between engineers, neuroscientists, medical doctors and patients is required. The most natural/intuitive control is one that is driven by neural signals tapped from the human central (CNS) or peripheral nervous system (PNS). In particular with the use of a neural interface directly connected to the PNS or CNS that is able to replace the sophisticated bidirectional link between the brain and the hand actuators and sensors, an advanced robotic limb might be able to put user intent into action and provide the user with perception of the hand itself by delivering sensory proprioceptive and exteroceptive information [5]. On this basis, the sensors to be endowed in advanced prosthetic hands should not be chosen and used just for closing automatic control loops (as in commercial devices), but also with the aim of delivering afferent information to the user through an adequate user-prosthesis interface (UPI).

One of the most challenging tasks in this field is certainly that of developing a dexterous intrinsic prosthetic hand, i.e. a hand that contains all its functional components (actuators, sensors, electronics, etc.), that can be used for patients after a distal transradial amputation. In the past decades several examples have been developed in research: the Sven hand, the Belgrade, the Southampton, the MARCUS, the TBM, the RTR II, the SPRING, the MANUS, the Ultralight hands in Forschungszentrum Karlsruhe, the Soft hand, the KNU hand [6-16]. Even if these prototypes differ in mechanisms, sensory equipment, performance and objectives, they all share the requirements of being low power, low weight, still allowing a number of prehension patterns useful in activities of daily living (ADLs). Such constraints were met by the use of different underactuated mechanisms (fundamental for reducing the number of actuators) and clutching systems (to save power once the grasp is stable): i.e. the two basic mechanical components in a prosthetic hand. All these intrinsic hands have been designed with the aim of being controlled by EMG surface electrodes or other intelligent control schemes [17], so that in most of them the sensorization is limited and mainly employed for the low-level control of the grasp. Even if some attempts to connect them to non-invasive feedback systems have been done, [18-20], most of these prototypes (with the exception of the Southampton-REMEDI hand, that contains sufficient active DoFs for different prehensile patterns, and an extended sensory system) would not be suitable if neurally interfaced with a large bandwidth link due to either their limited (or inexistent) sensorization or limited dexterity [21]. Other significant research even if related to extrinsic actuated hands to be used as research prostheses platforms include the CyberHand [5], the Yokoi hand [22], and Vanderbilt University prototypes [23,24]. In August 2008, researchers and companies supported by DARPA Revolutionizing Prosthetics Program (RPP) 2009 [25] have presented preliminary results at the Myoelectric Controls/Powered Prosthetics Symposium (MEC) held in Fredericton, NB, Canada. In particular: the RPP intrinsic hand [26], and a prototype from the Rehabilitation Institute of Chicago [27] were presented. Later, in May 2010 new prototypes or products from manufacturers were firstly exhibited at the ISPO (Intl. Society on Prosthetics and Orthotics) world congress held in Leipzig, Germany: in particular the Ottobock Michelangelo hand, the RSL Steeper Bebionic hand, and the second release of i-Limb, namely Pulse, from Touch Bionics.

The goal of this work was to design and develop a new, lightweight, dexterous, sensorized prosthetic hand with intrinsic actuation, i.e. a self-contained, transradial prosthetic hand able to be fitted in subjects with an amputation level, long below the elbow. This hand is meant to be clinically experimented by amputees employing different levels of interfaces, in order to investigate the effectiveness of more natural and intuitive control and feedback strategies. Interfaces under investigation will range from non-invasive EMG control and sensory substitution systems (as in [28,29]), to neural electrodes directly implanted into the PNS (as in [30]).

To this twofold aim a prototype with advanced integrated actuation and sensory features, compared to previous works and state of the art, was developed and successfully tested. This paper presents an overview of the design (partially covered in [21,31] and [32]) and focuses on the experimental characterization and discussion of the prototype performance, which is the unique contribution of this work. It demonstrates that due to its advanced embedded features and human-size, the SmartHand could be experimentally fitted on transradial amputees and employed as a bi-directional instrument for investigating -during realistic experiments- different interfaces, control and feedback strategies. Therefore the development of this hand opens up promising possibilities for the development of intuitive UPI and upper limb prosthetics in general.

## Requirements and Design Principles

In the design of a transradial prosthesis, the hand cannot be conceived to reproduce its human model where the hand is a non-separable part of the arm, deeply integrated with it (as in other designs like [5,22-24]), but must be considered like an independent, modular, self-contained end effector. Such requirement makes the robotic design a very challenging task that needs to be carefully addressed. *Functional *requirements for our design have been set according to interview results among the amputee community [33], and to the approximate percentage of utilization of the main grips in ADLs [34]. This hand should allow amputees to achieve:

1) Power grasps (used in approximately 35% of the ADLs);

2) Precision grasps (30%);

3) Lateral grasp (20%);

4) Index pointing (useful for pressing buttons, etc.);

5) Basic gestures (counting).

*Biologically *(i.e. in terms of bio-inspired design), the prosthesis should attempt to compare with the human hand in terms of:

1) anthropomorphism: i.e. size and weight (about 400 g), number and distribution of articulations, number and distribution of independently actuated DoFs;

2) static and dynamic biomimetism [35];

3) sensorization: types of sensors and distribution [2];

4) performance: speed (whole hand closing in less than 2 seconds) and grasping force (able to stably handle everyday objects).

**Figure 1:**
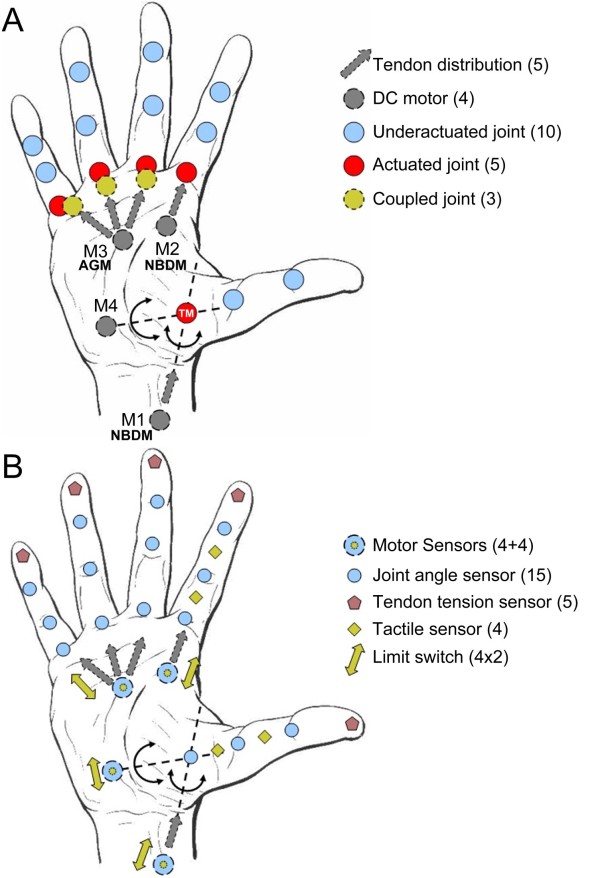
**SmartHand architecture**. Number of elements used are given in parentheses. A) Distribution of DoFs, tendons, joints and actuators. Abbreviations: NBDM, non-back-drivable mechanism; AGM, adaptive grasping mechanism; TM, trapezio-metacarpal joint; M1..4, motor 1..4. B) Distribution of sensors. Motor sensors are 4 current plus 4 position sensors.

The lack of high power density actuators yielded to design a device strongly based on underactuated and differential mechanisms. Figure 1A presents such architecture: there are 16 DoFs (three for each finger, plus one for the thumb abduction/adduction axis) and only 4 motors (i.e. 4 degrees of actuation, DoAs) that actuate five underactuated fingers based on Hirose's soft finger [36]. These are flexed just by a single tendon, and extended by torsional springs housed in the joints (as in the RTR II, and the CyberHand); their inherent differential mechanism allows all phalanxes to get in touch with the grasped object, allowing therefore, multi-contact stable grips. Fingers are operated using nylon coated steel tendons, pulleys and steel Bowden cables by 4 non-back-drivable actuation units based on DC motors located inside the palm. Thumb and index flexion/extension are independently actuated (by M1 and M2 in Figure 1A), whereas the middle, ring, and the little fingers are joined together (actuated by M3) by means of an adaptive grasping mechanism (AGM). The fourth motor (M4) is used for the thumb abduction/adduction movement, allowing different prehension patterns useful in everyday life. An actuation distribution like this one allows the hand to fulfil the functional requirements previously stated.

The hand sensory system is designed to be used both for the automatic control of the grasp (action) and for delivering sensory information to the user (perception) by means of UPIs with different degrees of invasiveness. In fact, recent studies have preliminarily shown the possibility of delivering force and position afferent information directly to the PNS, by means of an implanted neural interface [30,37], or touch, pressure and temperature sensations by employing a non-invasive sensory feedback system to redirected parts of the body after targeted reinnervation procedure [38]. More recently it has been shown how amputees can be made to experience a rubber hand as part of their own body by simply tricking their brain using the so-called *rubber hand illusion *[39]; a simple method based on a prosthesis equipped with tactile sensors for transferring sensations from the stump to the prosthesis was then outlined. Based on the reported studies, a sensor set that provides three different types of information, namely, position, tactile/pressure and force, was chosen. The spatial distribution of sensors in the hand (shown in Figure 1B) is similar to the natural concentration: higher on the independently actuated thumb and index fingers [40]. There are 32 proprioceptive and exteroceptive sensors based on traditional technologies embedded in the hand: 15 Hall effect-based angle sensors (integrated in all the finger joints), 5 strain gauge based tendon tension sensors (as in [5]) integrated in the fingertips (thus measuring the grasping force of each finger), 4 current sensors (one for each motor) and 4 optical tactile/pressure analog sensors (based on [41]) in the intermediate and proximal phalanxes of the thumb and index. Actuation units are also equipped with position sensors (either a resistive potentiometer or a digital encoder, measuring the released tendon length) and pairs of digital proximity sensors acting as limit switches (avoiding mechanical collisions). A detailed presentation of the SmartHand sensory system features is presented in [21].

## Robotic Hand Design

The hand is composed of a number of elements containing mechanisms, sensors and necessary electronics. Specifically it consists of: (i) 5 underactuated fingers, (ii) 2 capstan-based actuators driving the thumb and index flexion/extension (connected to M1 and M2), (iii) 1 adaptive grasping mechanism driving the middle-ring-little fingers (AGM connected with M3), and (iv) the thumb abduction/adduction mechanism (on M4). Each part is presented in detail in the following sub-sections.

### Underactuated Sensorized Finger

**Figure 2:**
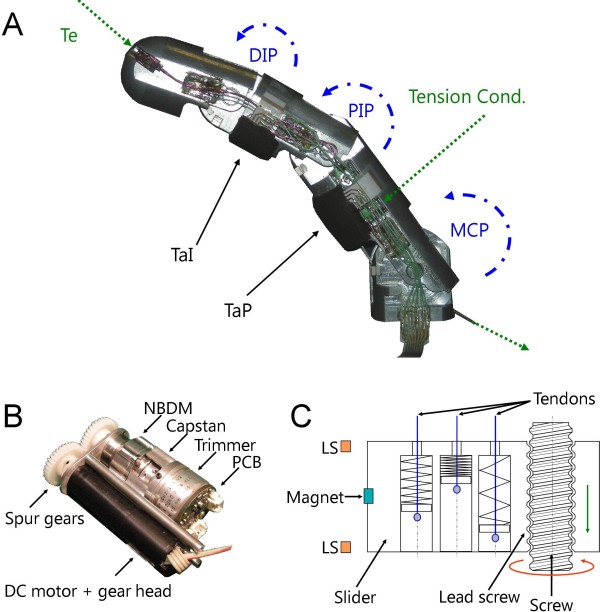
**SmartHand mechanisms**. A) Underactuated & sensorized finger; Te indicates the location of the tendon tension sensor; TaI and TaP are the intermediate and proximal phalanx tactile sensors; MCP, PIP and DIP are the hall effect joint sensors; Tension Cond indicates the location of the tendon tension sensor conditioning PCB. B) Capstan-based actuator C) Adaptive grasping mechanism scheme: the rotation of the screw (red arrow) causes the slider to translate (green arrow) due to the screw-lead screw coupling. The slider pulls (or releases) the three tendons simultaneously, which due to compressing springs allow for adaptation of the last three finger to the object. LS stands for limit switch sensor.

The fingers have an architecture based on Hirose's design [36] and are composed of 3 aluminium phalanxes (proximal, intermediate and distal) and 3 DoFs each (metacarpo-phalangeal joint, MCP, proximal-interphalangeal, PIP and distal-interphalangeal, DIP). The structure of fingers has been dimensioned according to anthropometric information available [42], in order to contain proprioceptive and exteroceptive sensors, as well as conditioning electronics and wires in a robust way. Finger components (springs and pulleys) were selected to allow the finger to replicate the human behaviour [35] while closing in free space; this feature enables the finger to correctly wrap around objects [5,43]. Figure 2A shows the index finger with all sensors embedded in it: joint angle, tactile and tendon tension sensors. The latter is composed of two parts: the strain gauge equipped micro-machined cantilever in series with the tendon stop (in the fingertip), and a small printed circuit board (PCB) containing the Wheatstone bridge and the instrumentation amplifier conditioning circuit (in the proximal phalanx). Miniature insulated wires soldered to the sensor pads and to the electronic boards, run laterally along the finger inside a hollow avoiding stretching (Figure 2A).

### Capstan-Based Actuators

In a majority of research projects on prosthetic hands, and in general in robotic hands, non-back-drivability is achieved by means of screw/lead screw pairs (as in all previously mentioned intrinsic hands with the exception of [16]). This is actually a simple to build mechanism but its efficiency is very low. To overcome such problem, a miniaturized high efficiency non-back-drivable mechanism (NBDM) based on wedge phenomena was developed for the SmartHand (described in detail in [44]).

For understanding this work, the NBDM should be simply regarded as a small-sized mechanical component (5900 mm3 volume, similar to a plastic bottle cap) that allows the transmission of the rotational motion, when it is originated by the motor shaft, stopping instead each motion that originates from the output shaft. The latter is connected to the driving capstan where the finger tendon is wound. The complete actuation unit (Figure 2B) from the input to the output is composed of:

1) a small-sized brushed DC motor (Faulhaber Minimotor, model 1319) with integrated planetary gear head (491:1);

2) a spur gear couple;

3) the developed NBDM;

4) a 6,5 mm radius capstan, which is finally connected to a commercial long rotational life resistive trimmer able to measure the released tendon length;

5) 2 Hall effect proximity sensors (Allegro MicroSystems Inc, model A3213) acting as limit switches, that together with a magnet fixed on one spur gear limit the tendon release.

A stainless steel microcable tendon is wound around the capstan, and runs into round steel wire spirals from the actuator output to the finger metacarpus. The system is completed by a round PCB topping the potentiometer (Figure 2B), containing filters for removing electrical noise from the position signal. Two identical actuation systems as the one described are housed in the palm and employed to independently actuate the thumb and index fingers flexion/extension movement.

### Adaptive Grasping Mechanism

This system is an improvement of the non-back-drivable adaptive grasping mechanism proposed by Massa et al. [11]. It drives the simultaneous flexion/extension of middle, ring and little fingers, as well as their adaptation to the object, allowing a stable, multiple contact grasp as in the natural hand. In this mechanism, the screw/lead screw could not be avoided: three tendons are connected to a linear slider by means of three compression springs (scheme in Figure 2C). The slider moves along the screw by means of a screw/lead screw pair and pulls (or releases) the tendons. Two limit switches are assembled along the screw, in order to limit the length of cable released. By means of the compression springs, during a general grasp, adaptation to the object of the three fingers is obtained: when the first finger comes in contact with the object, the relative spring starts to compress; the slider is free to continue its motion and the other fingers can flex reaching the object. If high forces are required, compression springs behave as a rigid link and the force is transmitted from the slider to the fingers. The actuation unit is composed of a brushed DC motor (Faulhaber Minimotor, Model 1331) with integrated planetary gear-head (reduction 16:1) and integrated encoder, a spur gear couple (reduction 1:2) and a non-back-drivable screw-lead screw coupling (pitch 0.7 mm) where the slider runs.

### Abduction/adduction Mechanism

**Figure 3:**
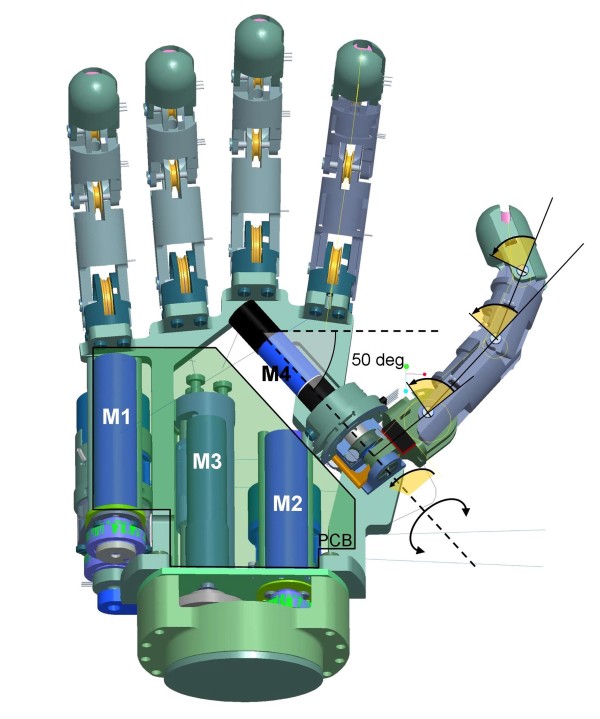
**The transradial prosthesis design**. Five fingers are actuated by means of four DC brushed motors M1-4 all located inside the palm structure. Motor M1 drives thumb flexion/extension, M2 drives index flexion/extension, M3 actuates the middle, ring and little fingers simultaneously and M4 actuates the thumb abduction/adduction. The thumb axis angle with regard to the horizontal plane is highlighted (50 deg). The main printed circuit board (PCB) is placed on the motors.

The thumb has two DoAs: one for flexion/extension, plus one for abduction adduction. With reference to the natural hand, the equivalent trapezio-metacarpal joint (TM in Figure 1A) has 2 DoFs. The hand is thus able to perform different prehensile patterns ranging from lateral to precision and power grasps. The flexion/extension metacarpophalangeal joint (Figure 3) is directly connected onto an extension of the brushed DC motor (Faulhaber Minimotor, model 1016) shaft; a certain degree of non-back-drivability is achieved by means of a high reduction (1024:1), this actually, allows slight adaptation of the thumb axis while it is closing against the other fingers in a precision grasp. The design is completed with two limit switches and the integrated encoder that measures the joint angle of adduction.

### Supporting Skeleton

All components of the hand are housed inside a supporting skeleton machined in Ergal alloy aluminium. The base of the skeleton integrates a standard myoprosthesis wrist adapter which includes a 4 wire bus for communication. The SmartHand control board (see section 3.6) is placed on the motors (cf. Figure 3), and covered by a plane carbon fibre plate acting as the palm of the hand.

### Flexible Controller & Communication

**Figure 4:**
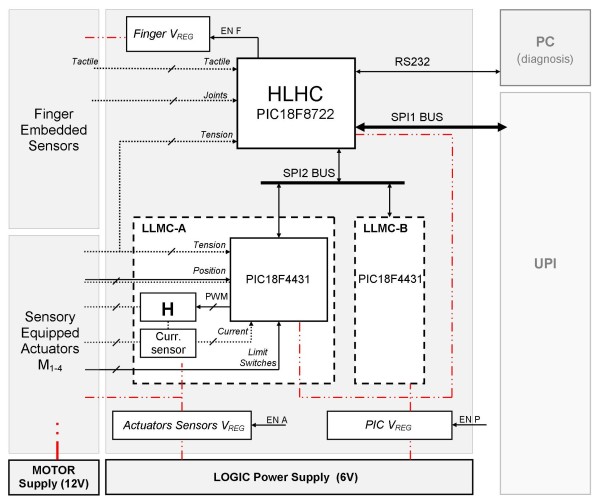
**The four axis control architecture based on microcontrollers**. Straight lines are logic lines; dotted lines are analog lines; dot-dot-dash lines are power supply lines. The high-level hand controller (HLHC) is directly connected to the external world both with a RS232 and SPI bus. The HLHC deals with the low-level motor controllers via an additional SPI bus. The LLMCs are directly connected to H bridge drivers (H) delivering current to the DC motors using pulse width modulation (PWM) technique.

The hand is integrated around the flexible electronic control architecture shown in Figure 4. The architecture has to be flexible enough to support the real time control of four active axes, real time identification of external commands, computation of control loops and delivering sensory biofeedback. A modular hierarchical architecture (as in [5,9,13]) based on a high-level hand controller (HLHC) and two low-level motor controllers (LLMCs) has been selected. Both LLMCs (LLMC-A and LLMC-B) are associated to two actuators whilst the host HLHC is in charge of the general functionality of the prosthesis. The HLHC, in master configuration, communicates through a fast serial peripheral interface (SPI) bus with the slave LLMCs, whereas external communication (UPI or a PC) with the HLHC can be handled by using a standard RS232 or (eventually) a fast SPI bus.

The main function of the internal software implemented in the LLMC is to provide all the necessary low level motor control functions (i.e. force control, position control modes, sleep mode). Therefore the microcontroller acts as a double finite-state machine (one for each motor) where the transitions between the different modes are triggered by HLHC commands coming from the SPI2 bus (Figure 4). The HLHC is in charge of sequencing LLMC functions to obtain meaningful operation of the hand (i.e. grasps and gestures) after an external command, to provide artificial sensory information to the UPI, to manage power modes and handle errors.

Current is delivered to the DC motors using integrated H-bridges and measured by a commercial current sensor (INA138, Texas Instruments). Two different power suppliers, one for the motors (12 V) and one for the controller (6 V) are needed. Since power budget is a key issue in prosthetics, particular attention has been paid to design a flexible architecture able to manage low power modes; three different voltage regulators are used. The first one, named as *PIC V*_*REG *_in the schematic of Figure 4, supplies the main components of the circuit, i.e. the three microcontrollers; its regulated output, is shut down by an external-world digital line (EN P). A second and a third regulator, named *Finger V*_*REG *_and *Actuators Sensors V*_*REG*_, are employed to supply the sensors embedded in the fingers and the sensory equipped actuators, respectively. These supplies, directly controlled by the HLHC (using EN F and EN A), may be used in switching modality when possible, in order to reduce power consumption. Moreover, the selected microcontrollers can operate in power-managed modes, thus saving energy.

A communication protocol for the RS232 bus based on a 115200 baud rate, has been developed. Basically it is a master-slave protocol where the hand behaves as a slave and the external world (either a PC for diagnosis or a UPI) as a master. Commands are divided into three main types: motor commands (for driving fingers in position or force control), sensor readings and automatic grasps. Without any particular firmware code optimization, all readings are served by the HLHC within a **measured delay **of 400 μs. This is a very short value that could allow implementing and closing control loops even by algorithms running on external systems. Internal control loops (position and force based on tendon tension) update errors every 1 ms (i.e. 4 independent and simultaneous loops running at a measured frequency of 1 KHz).

Automatic grasps are modelled on natural grasping. When an external unit (e.g. a control interface) invokes a grasping primitive (e.g. the lateral grasp), two different phases are sequenced by the HLHC: the preshaping and the grasping (closure) phase. After preshaping the desired finger tendon force is selected according to the grasping primitive. In the second phase, the prosthesis closes the involved fingers (in the lateral grasp example only the thumb closes) using tendon tension force control until the desired global tight force is reached. Both preshaping postures and desired grasping forces are set *a priori *and are based on the grasping primitive. For a detailed description of the automatic grasp controller refer to [45].

## Performance Analysis

Pictures of the SmartHand are presented in Figure 6, where a qualitative comparison with a traditional prosthesis and with the healthy hand of a transradial amputee are shown (a detailed comparison of the SmartHand with other research and commercial hands is presented in [21]). The weight of this hand is close to the natural hand weight and comparable to actual commercial prostheses: 530 g. This does not include the standard wrist attachment (145 g) and batteries (that are usually hosted in the prosthetic socket proximally to the residual limb). Performance with relation to the prosthesis requirements listed in the *Requirements and Design Principles *section and to practical usage have been evaluated. This comprehensive list of measured features includes: (i) finger dynamics, (ii) speed, (iii) grasping capabilities, (iii) grasping force, (iv) degree of adaptability, (v) supporting grasp capabilities and (vi) power consumption.

### Finger Dynamics and Speed

**Figure 5:**
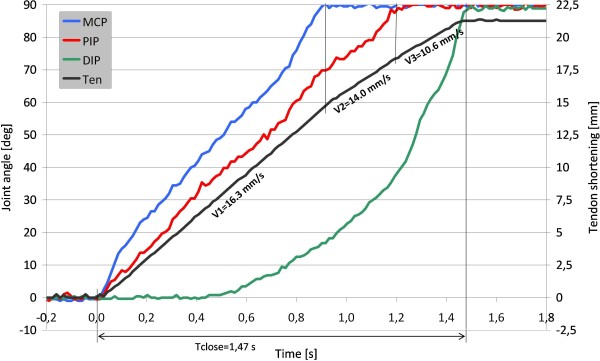
**Finger dynamics while closing at full speed**. Left Y axis: finger joint trajectories (metacarpo-phalangeal joint, MCP, proximal-interphalangeal, PIP and distal-interphalangeal, DIP, ranging between 0 and 90 degreees). Right Y axis: tendon shortening dynamics (*Ten *trace, ranging between 0 to 22 mm); superimposed on the graph are the maximum velocity (V1, V2 and V3) values, which are influence by the springs stiffness in the finger.

With the aim of experimentally evaluating the effective kinematics of the artificial finger in order to compare it with the natural model, a characterization has been done as follows. A simple C++ application was written to run on a PC (PC diagnosis in Figure 4), able to communicate with the hand controller by means of the communication protocol. This application was used to drive the index finger motor closure and to continuously monitor its MCP, PIP, DIP sensors and potentiometer sensors output. Sampling frequency was fixed at 70 Hz. The measured finger joint trajectories while the DC motor is driven at full speed are plotted in Figure 5: joint sensor dynamics (blue, red and green traces) are adjusted between 0 and 90 degrees (i.e. their effective angular range in degrees). The potentiometer value is rescaled in terms of tendon shortening (in millimeters): i.e. 0 mm when the finger is fully opened and 22 mm (i.e. the tendon stroke) if fully closed. The tendon shortening (Ten in Figure 5) highlights the varying maximum closing velocity (V1, V2 and V3) influenced by the springs stiffness in the finger.

The time plot in Figure 5 demonstrates some of the successfully achieved results from a prosthetic point of view:

1) as required, the finger kinematics is similar to the natural one while closing in free space (i.e. MCP joint speed is higher than the PIP one, and the DIP is the slowest; [35]), therefore the hand is able to perform stable grasps with multiple contact points [43];

2) minimum closing time is acceptable and comparable with commercial prostheses being only 1.47 s [44];

The speed slope (see the *Ten *curve) is divided in three parts basically based on which torsion spring counteracts the motor. Finally it must be noted that the four curves plotted in Figure 5 (Ten, MCP, PIP and DIP) are obtained using the communication protocol dealing with the hand controller in Figure 4. This demonstrates the successful operation of all developed components: the sensors, the acquisition electronics, the motor control, the communication protocol. For such measurement the low sampling frequency (70 Hz) was imposed by the C++ application developed on the external PC, not by the SmartHand controller; in theory actual limit of the sampling rate is about 2500 [Hz•Sample], or a sampling period of 400 μs. Similar measurements have been done for the other actuation units; minimum closing and opening times are reported in Table 1.

**Table 1:** Actuation units minimum closing and opening times

Actuator	Stroke	Closing time [s]	Opening time [s]
Index	22 mm	1.47	1.36
Thumb flexion	10 mm	0.67	0.62
Thumb abduction/adduction	90 deg	1.00	0.90
AGM	22 mm	1.53	1.27

### Grasping Capabilities

**Figure 6:**
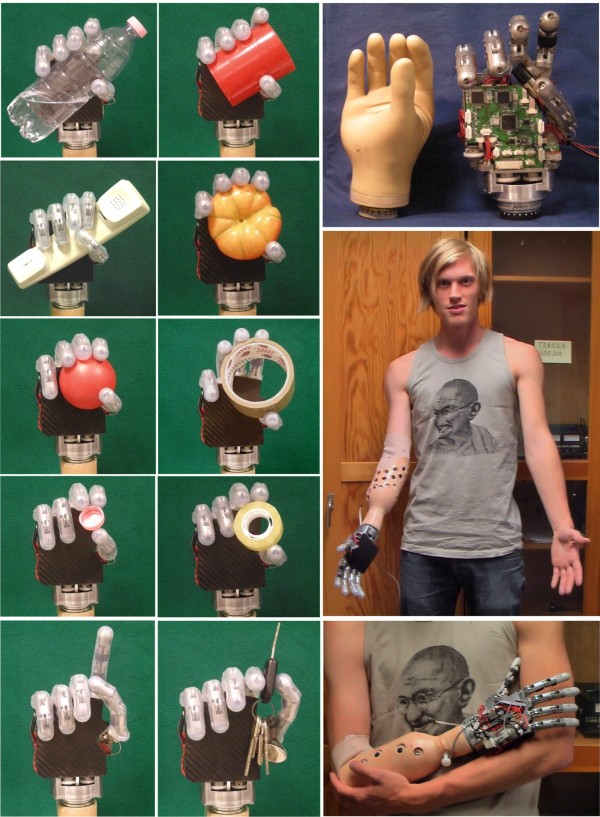
**SmartHand grasping capabilities**. Left columns: the SmartHand with silicone tubes on the fingers in the three basic prehensile patterns: power, precision and lateral grasps. Right column: SmartHand in comparison with a commercial prosthesis (SensorHand by Otto Bock, Austria) and fitted on a transradial amputee.

The hand is able to stably grasp many different objects performing the three basic prehensile forms. Pictures on the left side of Figure 6 show some of these grasps automatically achieved by the embedded controller using the automatic grasp control based on preshaping and closure phases described in the *Flexible Controller & Communication *section. From the receipt of the command the hand is able to achieve stable precision or power grasps in less than 2 seconds, and lateral grasps in less than 2.5 seconds (cf. time plots in Figure 9).

### Grasping Force & Degree of Adaptability

With the present underactuated, adaptive hand, stability in power grips is achieved by means of a multi-contact grasp differently from commercial prostheses where high forces are applied on few points [2]; in any case, the important issue for an amputee is the ability of stably grasping objects without slippage.

The maximum load that the hand could grasp in a power grasp prehensile form has been measured using the following setup. For all these measurements fingers were covered with silicone tubes (as those shown in Figure 6; hardness durometer 5 A) behaving as a cosmetic covering and improving friction between aluminum fingers and the (plastic) objects. A 36 mm diameter, 12 cm long, plastic (delrin) cylinder (180 g), connected by means of a steel cable to a 20 kg (full scale) mono-axial load cell, was grasped (at maximum strength) using the automatic grasp control. The hand was then switched-off (simulating a real life situation) and the cylinder was pulled out along the direction of its axis at a relatively high speed (about 130 mm/s), with the palm facing upwards, while the load cell signal was acquired (at 1 KHz) by a PCI data acquisition board. This procedure has been repeated 15 times and the mean load at which the cylinder starts to slip is about 16 N. Same measurements have been done with cylinders with larger diameter, representing objects handled in everyday life, 41 mm (225 g) and 71 mm (670 g) resulting in load force values of 36 N and 28 N respectively. Similarly, for the lateral grip (hand grasping a credit card) we measured 8 N (using this time a 2 kg load cell).

**Figure 7:**
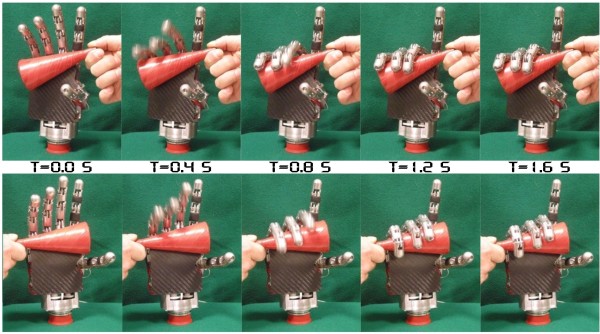
**Adaptive grasping mechanism**. Video sequences of the middle-ring-little fingers wrapping around a cone showing the degree of adaptability of the Adaptive Grasping Mechanism. In the top row the little is the first finger that touches the object, whereas in the lower row it is the last one (middle first). Superimposed on the pictures are the timestamps of the frames (in seconds).

The obtained values depend also on the transmitted torque from the actuators to the contact points. For the middle, ring and little fingers, this torque of course depends on the springs used in the AGM (cf. Figure 2C); more compliant springs will result in a higher degree of adaptability of the three fingers (with less transmitted torque), while stiffer springs will allow a higher torque transmission (but less adaptability). There is a trade off between maximum achievable grasp and adaptability. This trade-off needs to be taken into account in finding optimal values for spring stiffness. All the measurements (and pictures) reported in this paper have been performed using a fixed combination of springs (equal for each tendon with K = 2 N/mm) that allows a good degree of adaptability as shown by the video sequence in Figure 7. A delrin cone (h = 100 mm, d1 = 50 mm, d2 = 10 mm) is properly wrapped (i.e. three touching fingers) when grasped in both directions. The maximum tendon tension generated by the actuation units has been measured. Motor M3 connected to the AGM generates up to 35 N for each tendon; M1 (and the same for M2) connected to the NBDM may reach 45 N [46].

### Supporting Grasps

**Figure 8:**
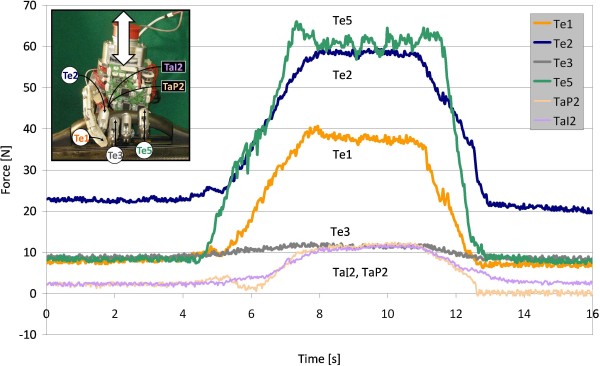
**Passive loading vs. sensory system**. Typical sensory output versus time when a 7.5 kg suitcase is lifted and released. Abbreviations: TaP, tactile proximal; TaI, tactile intermediate; Te, tendon tension sensors. Tactile sensors values are normalized in Newtons; Te4 is similar to Te3 and omitted for clarity.

Even if not able to apply large forces, a prosthesis should be strong enough to maintain high loads, for example carrying a suitcase: in such case the load would be applied on the mechanical structure of the fingers. Preliminary tests have been carried out: a 7.5 kg suitcase has been repeatedly lifted and released in quasi-static conditions, using the hand closed around the case handle (21 × 18 mm rounded rectangular section) as shown in the picture in Figure 8. Meanwhile the developed communication protocol was used to read data from the involved sensors. A typical sensory response for a lift/release cycle is shown in the time plot of Figure 8. Figures have been rescaled using the characteristics of the sensors. Four cable tension sensors (Te1, Te2, Te3, Te5) and two tactile sensors of the index finger (TaI2, TaP2) are plotted. Other sensors were not significantly involved in the task. Tactile sensors in the thumb did not touch; Te3 and Te4, have similar excursion and for a better readability only Te3 is presented. Sampling frequency was fixed at 35 Hz (again, limited by the external software). The graph is interesting as it shows how the load is shared among fingers: basically the little finger supports most of the load together with the index. The thumb, pressing on the dorsal side of the index (see picture) also contributes in supporting the handle from the left side, while the little supports it on the other side. In this particular case the middle and ring fingers are practically not loaded. This is possible when the AGM slider is completely closed and only the little finger spring is compressed (therefore no further adaptation is possible), and the other fingers do not touch.

The tension figures (all below 70 N) also give a qualitative indication on how far such a condition (7.5 kg load) is from tendon damage, that behaving as a mechanical fuse, represents a safety guard for the hand. Tendons are insured to up to 152 N; this means that the holding load is actually much higher than 7.5 kg. A 10 kg load was actually lifted with the same setup, resulting in no damage for the hand; more accurate stress measurements will be carried out in the future with a more specific setup, being this a non-trivial test to do.

By looking at the graph, some drift is seen in the sensor signals once the load is dropped; this is possibly due to a re-adaptation of the fingers around the handle while it was lifted, resulting in a different (lower energy) final static configuration of the joints. It is interesting to note that the tactile sensor values of the index finger after an initial phase are basically overlapped. This perfectly describes the inherent differential behaviour of the underactuated scheme adopted for the fingers, that tends to distribute torque among links.

### Power Consumption

**Figure 9:**
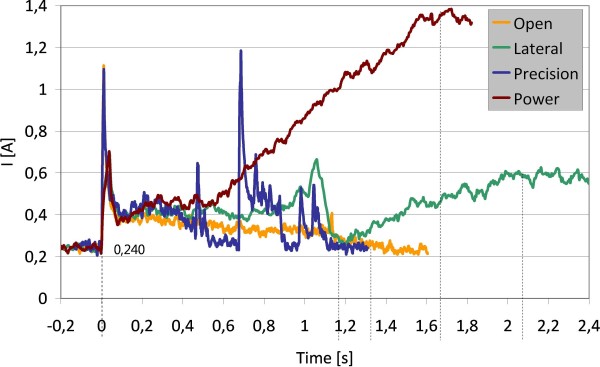
**Current consumption during grasps**. Typical power consumption during automatic grasps and opening. The traces have been shifted in order that movements start all at the same instant (t = 0s). For a better readability, traces have been discontinued once the movement ended and the current values were stable (indicated by the vertical lines).

Power consumption is one of the main requirements in prostheses design: the hand should work for a whole day without battery recharge. Plots in Figure 9 show the current demand of the SmartHand in three different extreme conditions implementing the automatic grasps commands: (i) maximum force lateral grasp, (ii) maximum force power grasp, (iii) opening from complete closure, and during (iv) a typical precision grip (the underactuated finger cannot generate high force precision grasps, as described in [28]). Current traces have been measured directly from the power supply using a current probe and captured by a digital oscilloscope sampling at 1 KHz, and then filtered (moving average, N = 10). Considering an amount of 3800 daily grasps, basically divided as in [34] (20% lateral, 50% power, and 30% precision grasps), and 3800 openings, a 1.22 Ah 12 V battery (for the motors) and a 1.22 Ah 6 V battery (for the control) would be sufficient for a full day autonomy, powering down the embedded controller when stable grasps are achieved. In fact when all sensors and peripherals are powered, electronics alone absorbs 240 mA (cf. Figure 9), i.e. 1.44 W. This is a significant value that should drop to mW levels (<100 mW) with the hand in sleep mode. The required charge is actually stored in quite small batteries that may be easily housed in the prosthetic socket. Current peaks and maximum values are sufficiently low and will not represent a problem for traditional (Nickel-Cadmium or Lithium-ion) rechargeable batteries.

## Discussion

The effective usability in domestic environments of innovative brain machine interfaces for prostheses control may be better evaluated (both functionally and cognitively) with tools that allow the user to interact with the environment itself rather than using virtual reality scenarios. To this aim, duplicating the complexity of the entire range of movements of the hand is hard enough provided infinite weight and size, but it needs to be accomplished within a slender morphology, replicating the look and the weight of the human hand. The *Anatomically Correct Testbed *(ACT) [47] and the commercially available *Shadow robot hand *[48], with their bulky actuation/control units, represent clear examples of such problem. In the constrained volume imposed by the biological model, trade-offs for approximating the natural hand are therefore mandatory. Several self-contained robotic prosthetic hands suitable for a transradial level of amputation have been developed up to now, nonetheless all of them would be rather inappropriate if/when a large bandwidth bi-directional link for control and perception would be available. Therefore, a successful integration of commercial components and state of the art techniques into a working anthropomorphic robotic hand still nowadays represents an open-problem in upper limb prosthetic research. The novelty of this prosthesis compared to previous prototypes and previous authors' work, is that it embeds in 530 g human-sized palm and fingers: (i) a set of non-back-drivable actuators allowing 85% ADLs grips, (ii) a sensory system comprising sensorial information perceivable by the patients using state of the art invasive or non-invasive interfaces [30,37-39], (iii) a low-power flexible controller able to drive actions and communicate in real time with external brain-machine interfaces. These combined features make the SmartHand prototype a unique robot/instrument for motor control and sensory feedback neuroscientific experiments with upper limb amputees.

Some aspects of the hand may be further discussed. Globally, the achieved mechanical, speed, and grip-power performance are considered satisfactory for the purposes under investigation. The maximum speed for example (closing time 1.5 s), is sufficiently high that patients would need significant training -aided by visual input- for matching the reaching movements of their limbs to the robotic hand speed. The 10 kg suitcase lifted by the hand, represents an interesting figure that robot hand designers should aim to. Regarding the grasping force, the hand was able to stably grasp objects without slippage; however while a 3.6 kg seems to be a sufficiently heavy object to maintain in a power grip prehensile form, an 800 g lateral grip could be inadequate for daily living activities (e.g. in the task of turning a key in a lock). Without replacing the actuation unit, such value may be improved by changing the mechanical architecture of the thumb from Hirose's configuration to a fixed kinematics scheme (as e.g. the Southampton hand finger). Regarding this point, it is important to underline that grasp stability strongly depends also on friction and compliance between materials, therefore, the use of a cosmetic glove would probably improve it. Regarding the weight (530 g) although similar to other multi-fingered commercial and research prostheses, it might become too heavy for the operator, especially once writs, batteries and cosmetic glove are added. The hand would be worn by the operator on the end of a closely fitting external socket (as shown in Figure 6), hence the weight would bear directly onto the skin of the stump. Since the lever-arm created is large the weight can obstruct blood flow in the underlying skin resulting in symptoms ranging from discomfort, to skin breakdown. Additionally heavy prostheses may cause damages to the wearer like elbow and shoulder overuse, rotary cuff problems, neck and back pain. For such reasons, if a more robust version of the SmartHand will be reengineered in the future, significant efforts should be spent to reduce its weight.

In the authors' opinion, robotic researchers should move towards the development of highly dexterous devices not only able to perform actions, but also ready to provide perception by means of a comprehensive artificial sensory system, in order to match the interface requirements and limits. Among the sensory sensations that may be transferred to the amputee by means of an adequate interface, joint position, touch and force sensors are included whereas only the temperature is not. Slippage and palm sensors are not included either but could be embedded in future versions of the hand. Even if currently there is no viable means to transfer all this afferent information to the amputee, guidelines for doing so have been traced [49], and novel -potentially revolutionizing- interfaces are nowadays currently being investigated [30,37,38]. To avoid the situation where the limiting component of the system is the artificial hand, whenever a novel large bandwidth interface will be ready for chronic clinical implantation, robotic research should be already prepared. It is also clear that for a robot, reliability depends on the number of moving parts and wires of the system, therefore a suitable compromise should be found based on the effective amount of information that can travel through the interface.

## Conclusions

The authors have presented the design and the performance evaluation of a 16 DoFs self-contained robotic hand to be used as a research tool for neuro-controlled upper limb prosthetics. Motion is generated by 4 brushed DC motors and transmitted to five underactuated fingers by means of non-back-drivable and differential mechanisms. Its actuation distribution allows the hand both to stably perform fundamental grips useful in activities of daily living, to independently point the index and counting. The fingers contain a total of 32 force, position and tactile sensors, and the hand hosts an internal control architecture able to plan grasps and to exchange with the external world proprioceptive and exteroceptive sensory signals. The weight is close to the natural hand weight and comparable to actual commercial prostheses (hand 530 g plus 145 g for the standard wrist attachment). Speed is comparable to commercial prostheses and slippage tests have shown that the hand is able to stably grasp with a cylindrical prehensile pattern and certain friction and geometric conditions up to 3.6 kg objects, nevertheless it is able to lift a 10 kg suitcase.

Future work will address the development of a cosmetic, protective glove in order to improve the aesthetic and grip of the hand. Functionalities will be tested in experimental sessions by amputees using UPIs with different levels of invasiveness, while the long term objective is to connect the hand directly to the human nervous system by means of neural electrodes in order to investigate the effectiveness of establishing an intuitive, bidirectional flow of information between the nervous system of the amputee and the robotic hand.

## Competing interests

CC and MC hold Prensilia Srl, the company that manufactures robotic hands as the one used in this work, under the license to Scuola Superiore Sant'Anna.

## Authors' contributions

CC and MC contributed to all the stages of this work (i.e., design of the hand, conducting experimental measurements and writing). MCC participated in designing the hand, as well as in writing. All authors read and approved the final manuscript.
